# Morphological and molecular characterisation of *Tristoma integrum* Diesing, 1850 (Monogenea, Capsalidae), including its complete mitogenome[Fn FN1]

**DOI:** 10.1051/parasite/2023016

**Published:** 2023-05-16

**Authors:** Romain Gastineau, Chahinez Bouguerche, Fadila Tazerouti, Jean-Lou Justine

**Affiliations:** 1 Institute of Marine and Environmental Sciences, University of Szczecin 70-383 Szczecin Poland; 2 Department of Zoology, Swedish Museum of Natural History Naturhistoriska riksmuseet Stockholm Sweden; 3 Université des Sciences et de la Technologie Houari Boumédiène, Faculté des Sciences Biologiques, Laboratoire de Biodiversité et Environnement: Interactions – Génomes BP 32 El Alia Bab Ezzouar, Alger Algérie; 4 Institut Systématique Évolution Biodiversité (ISYEB), Muséum National d’Histoire Naturelle, CNRS, Sorbonne Université, EPHE, Université des Antilles 57 rue Cuvier CP 51 75005 Paris France

**Keywords:** Monogenea, Monopisthocotylea, Capsalidae, Tristoma integrum, Mediterranean, Mitogenome, 28S

## Abstract

Capsalids are monopisthocotylean monogenean parasites found on the skin and gills of fish. Capsalines (subfamily Capsalinae) are large-sized capsalids, parasitic on highly prized gamefish, and species of *Tristoma* parasitise only the gills of swordfish (*Xiphias gladius*). We obtained specimens of *Tristoma integrum* Diesing, 1850 from swordfish collected off Algeria in the Mediterranean Sea. Here, we describe the specimens, including the key systematics characters of dorsolateral body sclerites. One specimen was used for a next generation sequencing analysis but a part of it, including the sclerites, was mounted on a permanent slide, drawn, and deposited in a curated collection. We characterised the complete mitogenome, the ribosomal cluster (including 18S and 28S) and additional genes such as Elongation factor 1 alpha (*EF1α*) and *Histone 3*. We also retrieved molecular information from the host tissue present in the gut of the monogenean and provide the sequence of the complete rRNA cluster of the host, *X. gladius*. The mitogenome of *T. integrum* is 13 968 bp in length and codes for 12 protein, 2 rRNA and 22 tRNA. Phylogenies of capsalids were generated from 28S sequences and concatenated mitochondrial protein-coding genes, respectively. In the 28S phylogeny, most subfamilies based on morphology were not found to be monophyletic, but the Capsalinae were monophyletic. In both phylogenies, the closest member to *Tristoma* spp. was a member of the *Capsaloides*. In an Appendix, we report the complex nomenclatural history of *Tristoma* Cuvier, 1817 and its species.

## Abstract (fr)

Les Capsalidae sont des monogènes Monopisthocotylea parasites de la peau et des branchies des poissons. Les Capsalinae sont des Capsalidae de grande taille qui parasitent les poissons de pêche sportive, très prisés, et les espèces de *Tristoma* ne parasitent que les branchies de l’espadon (*Xiphias gladius*). Nous avons obtenu des spécimens de *Tristoma integrum* Diesing, 1850 à partir d’espadons collectés au large de l’Algérie en Méditerranée. Nous décrivons les spécimens, y compris les caractères systématiques clés des sclérites dorsolatéraux du corps. Un spécimen a été utilisé pour une analyse de séquençage de nouvelle génération, mais une partie de celui-ci, qui comprend des sclérites, a été montée sur une lame permanente, dessinée et déposée dans une collection. Nous avons caractérisé le mitogénome complet, le cluster ribosomal (y compris *18S* et *28S*) et des gènes supplémentaires tels que le facteur d’élongation 1 alpha (*EF1α*) et *histone 3*. Nous avons également récupéré des informations moléculaires à partir du tissu de l’hôte présent dans l’intestin du monogène et fournissons la séquence du cluster d’ARNr complet de l’hôte, *X. gladius*. Le mitogénome de *T. integrum* a une longueur de 13 968 pb et code pour 12 gènes de protéines, 2 *ARNr* et 22 *ARNt*. Des phylogénies des Capsalidae ont été générées, respectivement à partir des séquences *28S* et des gènes codant pour les protéines mitochondriales concaténés. Dans la phylogénie *28S*, la plupart des sous-familles basées sur la morphologie n’ont pas été trouvées monophylétiques, à l’exception notable des Capsalinae. Dans les deux phylogénies, les membres les plus proches de *Tristoma* spp. étaient des espèces de *Capsaloides*. Dans une annexe, nous rapportons l’histoire nomenclaturale complexe de *Tristoma* Cuvier, 1817 et de ses espèces.

## Introduction

The Capsalidae are a family of monopisthocotylean monogeneans, characterised by a size larger than most other monopisthocotyleans, with several species over 1 cm in length, and a typical morphology with a large discoid haptor and two prominent anterior organs [59]. Among the Capsalidae, the subfamily Capsalinae includes about 60 species that parasitise the skin and gills of gamefish [18]. Chisholm & Whittington (2007) summarised the challenges of the modern taxonomist studying these parasites “First, over a third of the currently recognised capsaline species were described during the 1800s. Thus, not only can the original descriptions be difficult to obtain, but often no type material to verify these descriptions exists. Second, because capsalines are usually large and obvious parasites on cosmopolitan gamefish species, many parasitologists have collected them worldwide. Therefore, some capsalines from the same host species have been described as different species when collected at different localities globally. In these instances, it is highly likely that these capsaline species are synonymous” [18]. However, the large size of the capsalines is currently an advantage for the modern taxonomist because they provide enough DNA to allow molecular studies with routine methods, and are even large enough so that only a part of the body can be used (and destroyed) for molecular purposes, while the rest of the body can be kept on a permanent slide for morphological work and be deposited as a voucher (hologenophore) in a curated collection.

One of the genera of the Capsalinae, *Tristoma* Cuvier, 1817, was described as early as 1817 by Cuvier [19] with the description of *Tristoma coccineum* Cuvier, 1817. Diesing (1835) published a monograph on *Tristoma*, which reported four species, with only one of them (*T. coccineum*) still currently included in the genus [22, 23]. Another species, *Tristoma integrum* Diesing, 1850, was described later [24]. More than a century later, in 1968, Yamaguti [60] described two additional species, which Chisholm & Whittington (2007) considered as probably synonymous with the species above [18]. All species of *Tristoma* are parasites on the gills of the swordfish, *Xiphias gladius*, and are characterised by dorsomarginal sclerites in distinct transverse rows [18].

Because some capsalids have a pathological effect on fish of economic importance, and probably also because their large size makes it possible to obtain molecular data with relative ease, there are a number of sequences available [48], including several recently obtained complete mitogenomes [5, 61, 63, 65]. However, prior to this study, the genetic references for *Tristoma* were rather scarce. They consisted of eight sequences representing three partial genes: *28S*, Elongation factor 1 alpha (*EF1α*) and *Histone 3* [45, 48].

In a previous paper, we emphasised that many mitogenomes have been described, unfortunately, without any associated morphological study nor deposit of voucher material in a collection (Table 3 in Ayadi *et al.*, 2022 [2]). The aim of this work was to provide morphological arguments for correct identification of our material and associate it with as much molecular information as possible, including on the host.

In this study, we redescribe *Tristoma integrum* based on material collected in the Mediterranean Sea off Algeria, and we characterise its complete mitogenome, the complete ribosomal gene cluster including *28S* and *18S*, and additional genes including Elongation factor 1 alpha (*EF1α*) and *Histone 3*. The molecular work was performed on a specimen for which a lateral part of the body, showing the characteristic lateral sclerites, was mounted on a permanent slide and deposited in a museum collection, therefore allowing traceability; the sclerites were drawn to ascertain the specific identity. In addition, we could retrieve the DNA from the host from the digestive tract of the monogenean, which was used for the molecular analysis, therefore ascertaining the host identity with molecular tools.

In an Appendix, we provide details of the historical account of *Tristoma* and *Tristoma integrum*, their intricate nomenclature, and our chase for the original description of the species.

## Material and methods

### Collection and sampling of fishes

From 2017 to 2018, gills of 10 dead specimens of *Xiphias gladius* were collected directly from local fishermen in Bouharoun (36,370 N, 2,390 E) and Cap Djinet (36,877 N, 3,720 E). Fish specimens were processed shortly after capture and morphologically identified in the field using keys [28]. Gills were put separately in isolating bags, transferred to the laboratory in an ice bag, and examined at the laboratory on same day. The gills were cut into several parts, placed in Petri dishes in seawater, and observed under a stereomicroscope (Carl Zeiss™ Stemi™ DV4 Stereomicroscope, Oberkochen, Germany) for monogeneans. Synonyms, and scientific and common names of fishes are those provided in WoRMS [58] and FishBase, respectively [29].

### Morphological methods for monogeneans

Monogeneans were removed from the gills using dissecting forceps. Monogeneans were heat-killed with freshwater then preserved in 70% ethanol, stained with acetic carmine, dehydrated in a graded series of alcohol (70, 96 and 100%) for 15 min each, cleared in clove oil, and finally mounted in Canada balsam. Monogeneans were identified on stained whole mounts.

Whole stained mounts were photographed using a Zeiss microscope equipped with camera.

**Figure 1:**
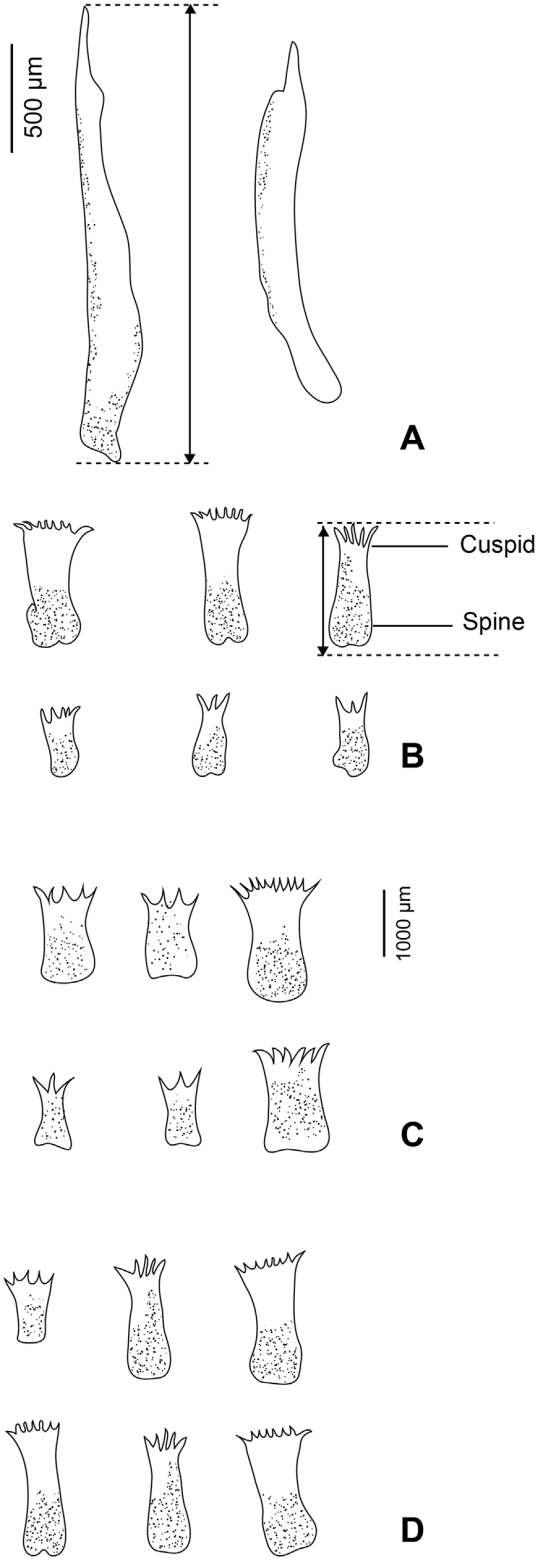
Sclerotised parts of *Tristoma integrum* Diesing, 1850 from *Xiphias gladius*. A, hamuli; B–D, dorsomarginal spines. A, B, specimen SMNH-209004; C, specimen SMNH-209006 (whole morphology in Fig. 2); D, specimen SMNH-209003, hologenophore. Note that dorsomarginal spines of the hologenophore are similar to the specimen drawn whole.

Drawings were made with a Nikon Eclipse i80 microscope with DIC (differential interference contrast) and a drawing tube. Drawings were scanned and redrawn on a computer with Adobe Illustrator. The sclerotised structures (dorsomarginal spines and hamuli) were measured according to Figure 1. Measurements are in micrometres, and indicated as means and between parentheses, the range and number of measurements. The nomenclature of internal anatomy and sclerotised marginal body structures used by Barse & Bullard (2012) for *Capsala laevis* (Verrill, 1875) is adopted here [7].

### Deposit of specimens

Voucher specimens of monogeneans were deposited in the Swedish Museum of Natural History, Stockholm, Sweden (Naturhistoriska riksmuseet), under registration numbers SMNH 209003–209011. Fish specimens were not deposited.

### Traceability of specimens used for molecular methods

For complete traceability of the molecular study, special care was taken to ensure that hosts and monogeneans were labelled with respect to host-parasite relationships [1–4, 11–15, 17, 33]. For one monogenean specimen (SMNH 209003), a lateral part of the body containing marginal spines was separated with a scalpel and mounted on a permanent slide and deposited as SMNH 209003, and the rest of the body was submitted to molecular analysis (and thus destroyed); this specimen is therefore a hologenophore according to Pleijel *et al.* (2008) [49].

### Next generation sequencing, assembly, data-mining and annotation

The SMNH 209003 specimen was sent to the Beijing Genomics Institute sin Shenzhen, China. The institute took care of the DNA extraction and performed sequencing on a DNBseq platform, from which ca. 40 million clean 150 bp paired-end reads were obtained. Reads were deposited on Sequence Read Archive (SRA) and are available as part of the BioProject PRJNA947900 (https://www.ncbi.nlm.nih.gov/sra/PRJNA947900). Assemblies were performed with SPAdes 3.15.5 [6] with two different k-mer of 85 and 125. Contigs of interest were extracted by data mining using blastn command-line [9], using as references the sequences indicated in Table 1. After circularisation and trimming, the mitogenome was annotated with the help of MITOS [8] and manually curated.

**Table 1:** List, accession number and references of the sequences used for customised blastn analyses.

Target sequence	Sequence used as database	Species and sequence used as database	Reference
Mitogenome of *T. integrum*	MN746369	*Capsaloides cristatus* (complete mitogenome)	[61]
Nuclear rRNA of *T. integrum*	AF131715	*T. integrum* (partial *28S* gene)	[45]
Histone 3 gene of *T. integrum*	FJ972130	*T. coccineum* (partial *Histone 3* gene)	[48]
EF1α gene of *T. integrum*	FJ972071	*T. coccineum* (partial *EF1α* gene)	[48]
Nuclear rRNA of *X. gladius*	DQ533143	*X. gladius* (partial *28S* gene)	[54]
Mitogenome of *X. gladius*	AP006036	*X. gladius* (complete mitogenome)	[44]

The position of the tRNA was verified using Arwen, with the -gcflatworm option [39]. The map of the mitogenome was obtained from the OGDRAW portal [40]. The annotation of the clusters of nuclear rRNA genes for both the parasite and its host were done with the help of Rfam [34].

### Phylogeny

Two different phylogenies were performed. The first one was a multigene phylogeny performed on concatenated amino acid sequences of the mitochondrial proteins from the available mitogenomes of Capsalidae. The second was based on the dataset of partial *28S* gene sequences from Perkins *et al.* [48] and Mollaret *et al.* [45]. For the multigene phylogeny, protein sequences were extracted and aligned separately using MAFFT 7 [36], trimmed using trimAl with the – automated1 option [16], and alignments were then concatenated using Phyutility 2.7.1 [53]. For the *28S* inferred phylogeny, partial genes were aligned with the complete gene from *T. integrum* SMNH 209003 using the Clustal function of MEGA X [38] and trimmed manually at the endings. Evolutionary models were chosen according to ModelTest-NG v0.1.7 with default parameters [20]. All phylogenies were conducted using IQ-TREE 2.2.0 [43] with 1 000 ultrafast bootstrap replications.

## Results

### Short redescription of *Tristoma integrum* Diesing, 1850 (Figs. 1–4)

*Synonyms:* Dawes (1947) listed the following synonyms [21]: *Tristoma coccineum* Cuvier, 1817, in part; *T. coccineum* Cuvier of Taschenberg, 1879; *T. rotundum* Goto, 1894.

*Type-host*: *Xiphias gladius*, the Swordfish (Xiphiidae) [24].

*Possible additional hosts*: *Tetrapturus belone* Rafinesque (Istiophoridae), the Mediterranean spearfish [47]; *Mola mola* (Linnaeus) (junior synonym of *Mola rotunda* Cuvier) (Molidae) [47].

*Type-locality*: off California [24].

*Additional localities*: Northwest Atlantic [18, 30, 31, 51]; Mediterranean Sea, off France and Italy [27, 41, 42, 47, 56]; off Turkey [46]; off Algeria [52], this paper.

*Specimens from Algeria*, *from the gills of Xiphias gladius*. Vouchers deposited in the collection of the Swedish Museum of Natural History, Stockholm, Sweden (Naturhistoriska riksmuseet), SMNH 209003-209011. Hologenophore [49], lateral part showing marginal spines mounted on slide, the rest of body part cut off and used for molecular analysis, SMNH 209003.

*Site on host*: Gills.

Description of specimens from *Xiphias gladius* from Algeria. Based on 10 specimens (9 whole specimens + hologenophore). Measurements in Table 2.

**Table 2:** Measurements of *Tristoma integrum.*

Species	*Tristoma integrum*	
Host	*Xiphias gladius*	
Locality	Off USA, Western Atlantic	Off Algeria, Western Mediterranean
Source	Price, 1939 [51]	Present study
	2	9
Body length	575–7000	580–7650
Body width	600–650	4900–6180
Number of spines per row	6	6
Number of cuspids per spine	3–5, usually 4	3–7
Spines length	20–26	18–25
Ventral sucker length	850–935	810–930
Ventral sucker width	765–850	780–845
Pharynx *	510	810–880
Haptor *	1400–1600	1250–1450
Number of haptoral septa	7	7
Large hooks length	110–133	75-95
Ovary length	255–370	210–420
Ovary width	680–765	650–810
Distance ovary-pharynx	400	396–460
Egg length	–	–
Egg width	90	–

*refers to diameter.

**Figure 2:**
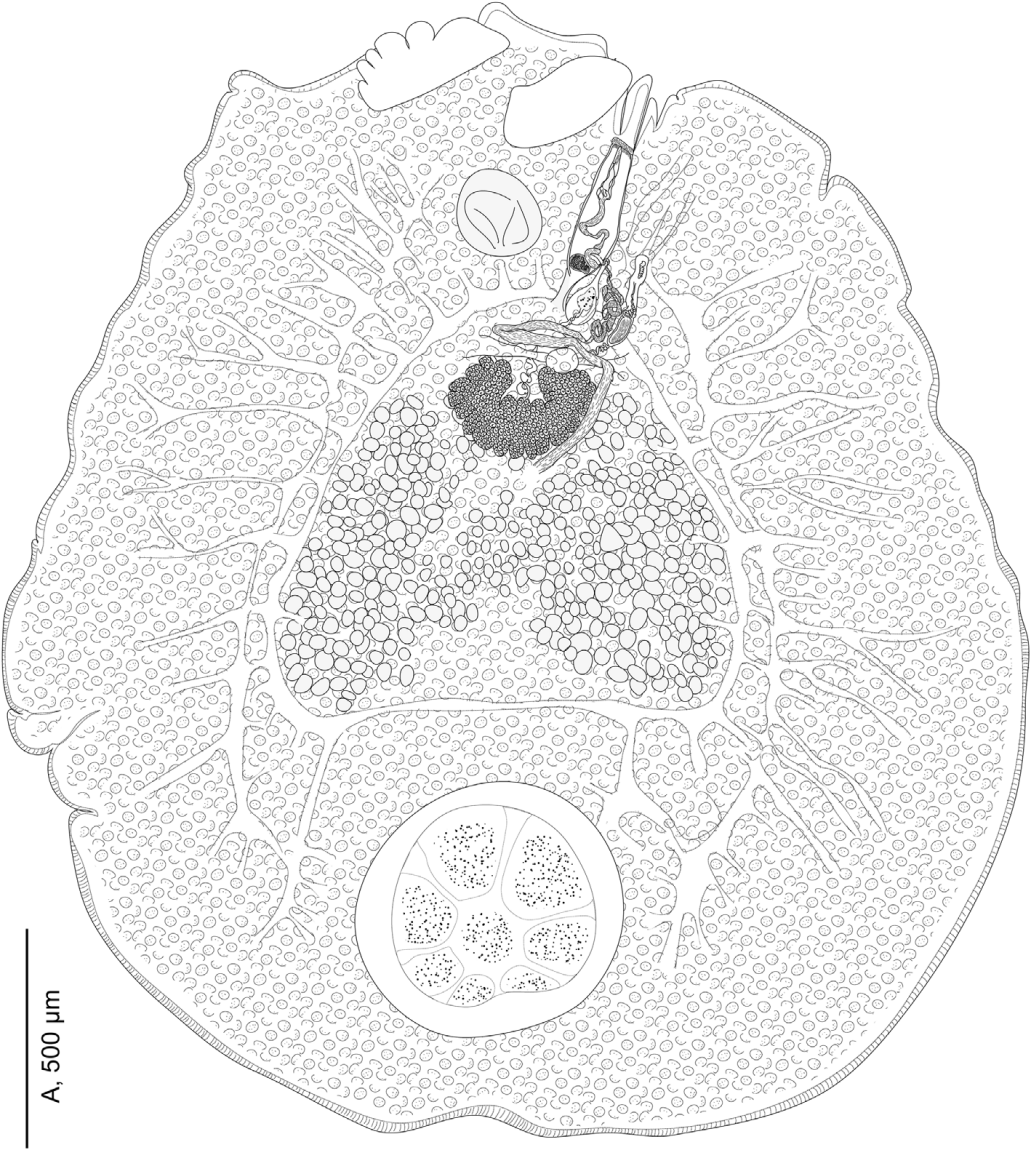
*Tristoma integrum* Diesing, 1850 from *Xiphias gladius*. Specimen SMNH-209006, whole morphology, ventral view. Hooklets not represented.

Body oval to rounded (Fig. 2), armed dorsally on its lateral margins with numerous transverse rows of marginal spines; each containing from 2 to 4 spines. Spines somewhat similar, 3 to 13 cuspids per spine (Fig. 1). Ventral body surface lacking papillae on most of its surface, but posterior body part covered ventrally with visible prominent papillae. Haptor circular, sucker-like, with a visible marginal membrane and a pair of haptoral anchors (Fig. 1). Haptor divided by seven radial weak septae into seven marginal more or less triangular loculi and a central one. Prohaptoral suckers oval to elliptical. Foureye spots. Mouth aperture transversal (Figs. 4A, 4C). Pharynx ovoid (Figs. 4A, 4C), muscular, with a posterior constriction. Intestinal branches with numerous diverticula, confluent posteriorly; intestinal diverticula reaching lateral margins and united in interintestinal field.

**Figure 3:**
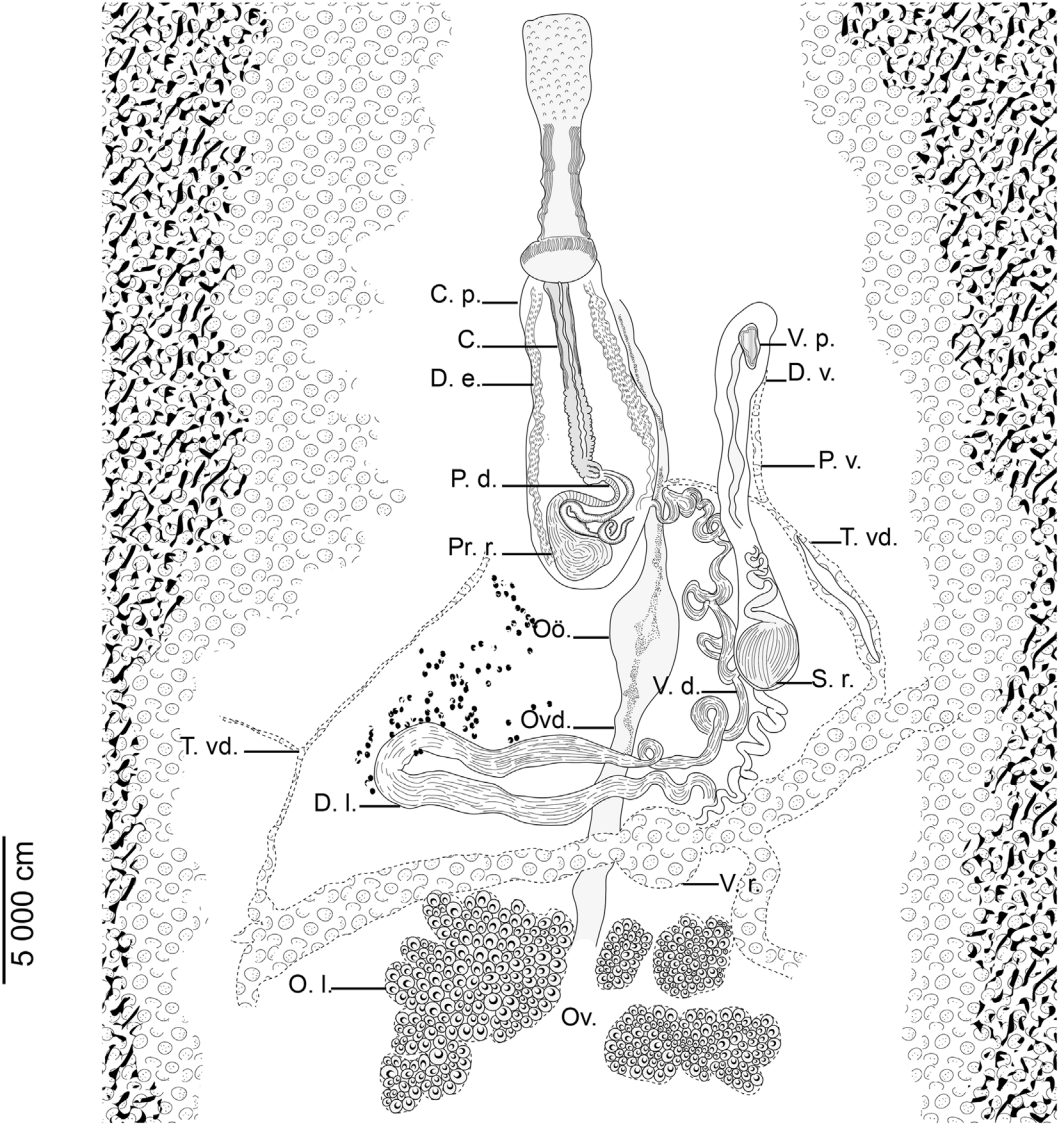
*Tristoma integrum* Diesing, 1850 from *Xiphias gladius*. Anatomy of reproductive organs, ventral view. C.: cirrus. C.p.: cirrus pouch. D.e.: ductus ejaculatorius. D.l.: distal loop of vas deferens. D.v.: distal vagina. O.l.: ovary lobes. Oö.: oötype. Ov.: ovary. Ovd.: oviduct. P.d.: prostatic duct. P.v.: proximal vagina. Pr.r.: prostatic reservoir. S.r.: seminal receptacle. T. vd.: transverse vitelloduct. V.d.: vas deferens. V.p.: vaginal pore. V.r.: vitelline reservoir.

**Figure 4:**
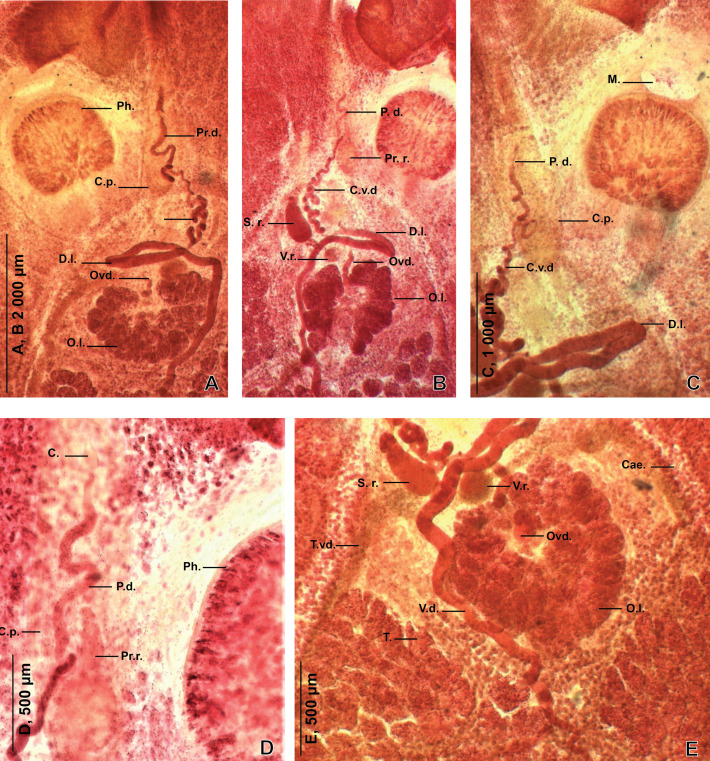
*Tristoma integrum* Diesing, 1850 from *Xiphias gladius*. Photographs: Details of reproductive organs, ventral view. Specimens SMNH-209005-08. C.: cirrus. C.p.: cirrus pouch. C.v.d.: coiled ascending portion of vas deferens. Cae: caeca. D.l.: distal loop of vas deferens. M.: mouth. O.l.: ovary lobes. Ov.: ovary. Ovd.: oviduct. P.d.: prostatic duct. Ph.: pharynx. Pr.r.: prostatic reservoir. S.r.: seminal receptacle. T. vd.: transverse vitelloduct. V.d.: vas deferens. V.r.: vitelline reservoir.

Testes varied in shape and dimensions (Fig. 4E), confined to area between intestinal crura. Vas deferens ventral to ovary, originating somewhere at level of anterior testes (Fig. 4E), passing anteriorly on marginal part of ovary; folding anteriorly to vitelline reservoir then strongly winding before entering cirrus sac and uniting with prostatic duct (Figs. 4A–4C). Cirrus sac oval and elongated (Figs. 4A, 4C, 4D), thick walled with visible longitudinal muscles) (Fig. 3). Cirrus sac containing cirrus and accessory gland reservoir (prostatic reservoir) (Fig. 4D). Accessory gland reservoir (prostatic reservoir) fusiform, located at the base of cirrus pouch (Fig. 3). Cirrus cylindrical, muscular, thick walled, papillate for or half of its length (Fig. 3); ejaculatory duct thin, well visible (Fig. 3). Genital pore ventral, at midlevel of pharynx. Ovary rosette shaped, voluminous and lobed (Figs. 4A, 4B, 4E). Oviduct originating at level of central lobe of ovary (Figs. 4A, 4B, 4E). Junction between oviduct and vitelline reservoir not observed. Uterine aperture weak, posterior to genital atrium. Oötype fusiform; shell gland ducts convergent at midlevel of oötype (Fig. 3). Vagina remarkably long, funnel shaped, containing vaginal duct and a seminal receptacle. Vaginal duct parallel to uterus; distal portion of vaginal duct tubiform, long and straight; proximal portion of vaginal duct somewhat oval; distal portion of vagina projecting in vaginal opening (Fig. 3). Seminal receptacle rounded to oval (Figs. 4B, 4E), immediately posterior to proximal portion of vaginal duct, connected to vitelline reservoir by a narrow-convoluted duct (Fig. 3). Vaginal opening dextral, ventral, posterior to male genital pore, with a visible glandular wall. Vaginal wall with several layers. Vitellarium including vitelline follicles and two sorts of ducts: lateral ducts and transverse vitelline ducts (Fig. 4E). Vitelline follicles extensive, abundantly developed along intestinal branches and occurring as well in spaces between testes. Transverse vitelline ducts large; lateral ducts smaller, reaching lateral body margin. Vitelline reservoir rounded (Fig. 4E), well visible, posterior to transverse loop of vas deferens (Fig. 3). Ovo-vitelline duct and eggs not observed.

*Lateral sclerites*: drawn in three specimens (Fig. 1): the specimen figured as a whole mount (SMNH-209006), the hologenophore (SMNH-209003) and another specimen (SMNH-209004); lateral sclerites were identical in all these specimens. Each row of marginal spines comprising from 2 to 4 spines. Spines similar in size, varying in number of cuspids per spine. Innermost spines with 3 to 7 short cuspids. Outermost spines comb-like, with 8 to 13 short cuspids. No 1-cuspid spines whatsoever.

### The mitogenome of *Tristoma integrum*

**Figure 5:**
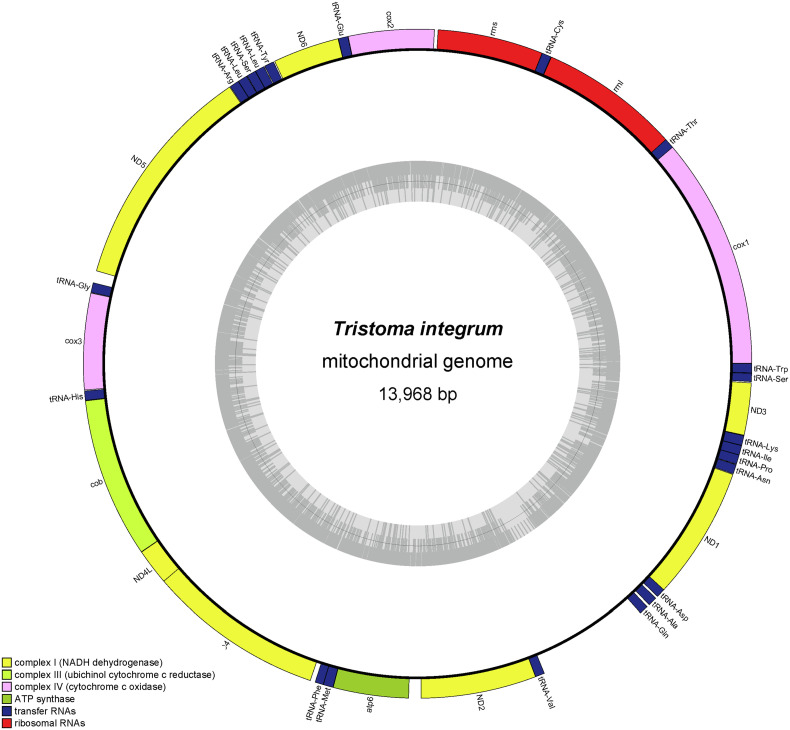
The mitogenome of *Tristoma integrum* (specimen SMNH-209003). The mitogenome includes 12 protein-coding genes, 2 rRNA and 22 tRNA, and is 13 968 bp long.

A 14 003 bp long contig with a coverage of 64.7× was retrieved after assembly (k-mer 125). Its AT-rich extremities showed redundancies, but also contained repeated sequences. It was trimmed to a circular contig of 13 968 bp, but due to the aforementioned presence of repetitions, this size should be taken with care. The mitogenome (GenBank Accession Number OQ355698) contains 12 protein-coding genes, 2 rRNA and 22 tRNA (Fig. 5). It was not possible to find a stop codon for the *cox1* gene, which would overlap tRNA-Thr by 9 bp if terminated by a natural stop and showed no obvious clue of a premature termination by A residues to the mRNA. The *cox2* gene is terminated by the presence of *tRNA-Glu* and the addition of A residues to the mRNA.

### Multigene phylogeny and comparison with the mitogenomes of other Capsalidae

**Figure 6:**
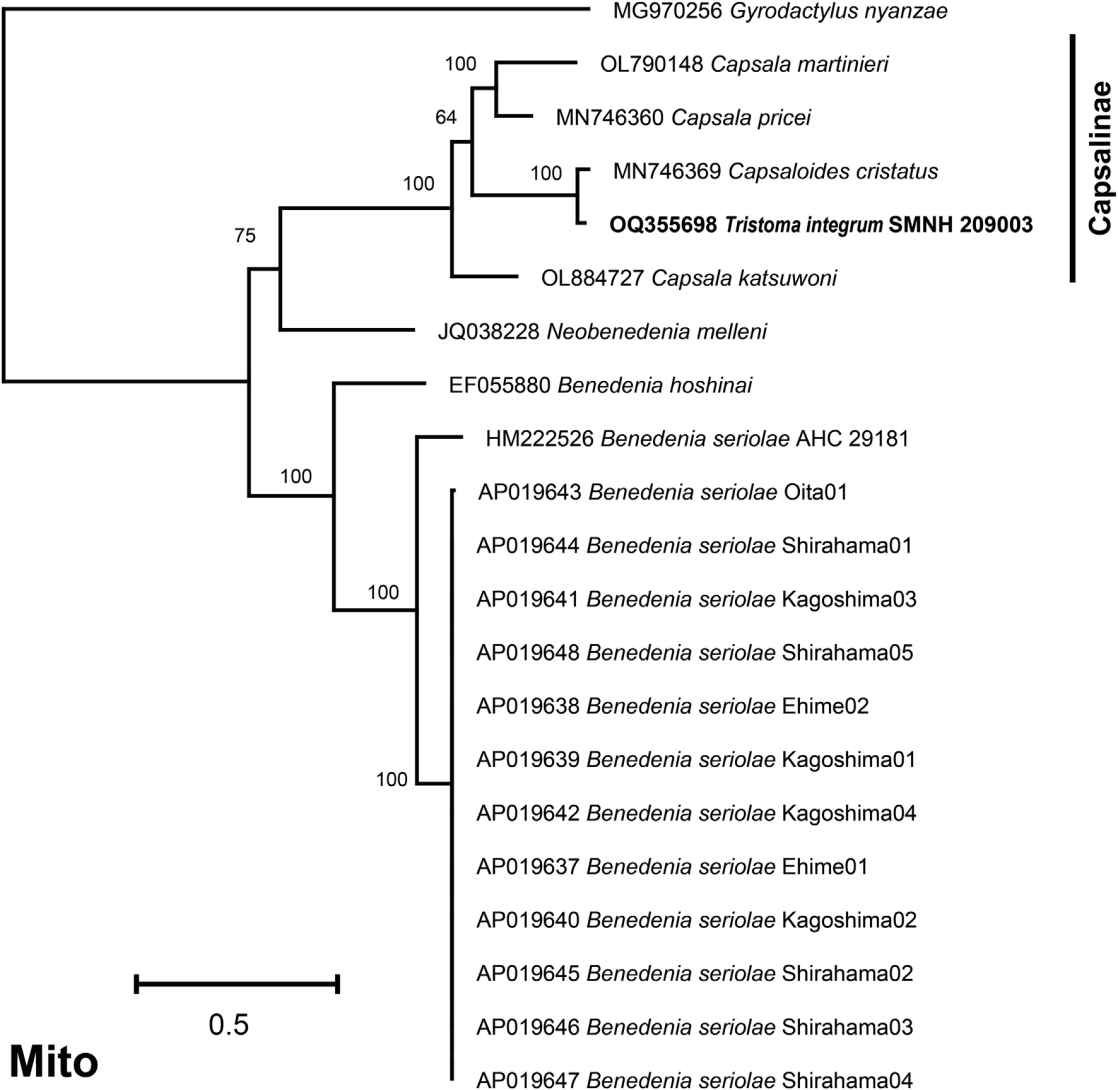
Maximum likelihood phylogenetic tree (MTZOA+G4+F model) obtained from concatenated amino-acid sequences of the mitochondrial proteins of *Tristoma integrum* and other Capsalidae. The tree with the best likelihood is shown and ML bootstrap support values are indicated. The scale indicates the number of substitutions per site. The limits of the subfamily Capsalinae is indicated on the right.

The best model of evolution returned by ModelTest-NG was the MTZOA+G4+F model. The multigene phylogeny associated *T. integrum* with *Capsaloides cristatus* Yamaguti, 1968 with maximum support at the nodes (Fig. 6). Both species were comprised of a bigger cluster that contained *Capsala martinieri* Bosc, 1811, *Capsala pricei* Hidalgo-Escalente, 1958 and *Capsala katsuwoni* (Ishii, 1936) Price, 1938; all these are members of the Capsalinae.

In Table 3, we compare the different features observed in the mitogenomes of the species belonging to the cluster highlighted in the phylogeny. It is worth noting that *T. integrum* and *C. cristatus*, which appear as sister species in the phylogeny, are also the only taxa sharing a putative overlap, of similar length, between *cox1* and *tRNA-Thr*. However, possibly for this reason, the annotation of the *cox1* gene of *C. cristatus* was noted as unverified in GenBank. The overlap between *ND4L* and *ND4* seems to be a constant feature. *Neobenedenia melleni* (MacCallum, 1927) Yamaguti, 1963, which appears slightly outside the cluster mentioned above but clearly distinct from *Benedenia* spp., also displays this 28 bp *ND4L*/*ND4* overlap [65]. Conversely, neither *Benedenia hoshinai* Ogawa, 1984 [35] nor any of the specimens of *Benedenia seriolae* (Yamaguti, 1934) Meserve, 1938 [37, 48] harbour any such overlap. However, there is an intergenic sequence of ca. 118 bp between *ND4L* and *ND4* in specimens of *B. seriolae* and this intergenic sequence is 282 bp in length in *B. hoshinai*.

**Table 3:** Comparison of the mitogenomes of Capsalidae directly clustering with *Tristoma integrum*, with accession number, size (in bp), references and presence/absence of the peculiar features detected.

Name	GenBank	Size (in bp)	Putative overlap between *cox1* and *tRNA-Thr*	Overlap between *ND4L* and *ND4*	Other features	Reference
*Tristoma integrum*	OQ355698	13 968	Yes (9 bp)	Yes (28 bp)	Premature stop of *cox2*; *ND4* and *ND4L* start with a GTG	This study
*Capsaloides cristatus*	MN746369	13 948	Yes (10 bp)	Yes (28 bp)	*ND4* starts with a GTG; start codon not found for *ND4L*	[61]
*Capsala pricei*	MN746360	13 851	No	Yes (28 bp)	*ND2* and *ND4L* start with a GTG	[63]
*Capsala martinieri*	OL790148	13 984	No	Yes (28 bp)	*ND3* starts with a GTG; *ND4* starts with a TTG	[62]
*Capsala katsuwoni*	OL884727	13 265	No	Yes (28 bp)	*ND2*, *ND4*, and *ND4L* start with a GTG	[62]

**Table 4:** Molecular information available for *Tristoma* spp. before our study. All available sequences provided by Perkins *et al.*, 2009 [48].

Species	Genetic marker	GenBank	Locality
*Tristoma coccineum*	Ribosomal RNA for the large subunit *28S* rRNA	FJ972014	Mediterranean
	Histone *H3* gene	FJ972130	Mediterranean
	Translation elongation factor *EF1α*	FJ972071	Mediterranean
*Tristoma integrum*	Ribosomal RNA for the large subunit *28S* rRNA	FJ972015	Mediterranean
	Histone *H3* gene	FJ972131	Mediterranean
*Tristoma* sp.	Ribosomal RNA for the large subunit *28S* rRNA	FJ972016	Atlantic Ocean
	Histone *H3* gene	FJ972132	Atlantic Ocean

### The cluster of ribosomal RNA genes of *Tristoma integrum* and *28S* inferred phylogeny

A 6 273 bp long contig containing the cluster of rRNA genes was found with a high coverage of 805.91x. After verification on Rfam and trimming, the complete cluster was 5 839 bp long (GenBank: OQ349751), distributed as 1 985 bp (*18S*), 462 bp (*ITS1*), 153 bp (*5.8S*), 332 bp (*ITS2*), and 2 907 bp (*28S*). The *28S* gene was compared with the partial *28S* genes already available in GenBank for *T. integrum*. Our sequence of *Tristoma integrum* was found to be 99.02% identical with FJ972015 (409 bp from *X. gladius* collected off Italy [48]) and only 96.66% with AF131715 (329 bp from *X. gladius* collected off France [45]). However, in the case of AF131715, most differences were found in the 3′ extremity of the sequence and were not shared with FJ972015, and are likely to be errors from early (2000) manual Sanger sequencing. After trimming of the dubious 3 ending, the percentage of identity rose to 99.68% between *T. integrum* SMNH 209003 and AF131715, and 98.80% with FJ972015. It is worth noting that all polymorphisms were found in the highly variable D1 divergent domain.

**Figure 7:**
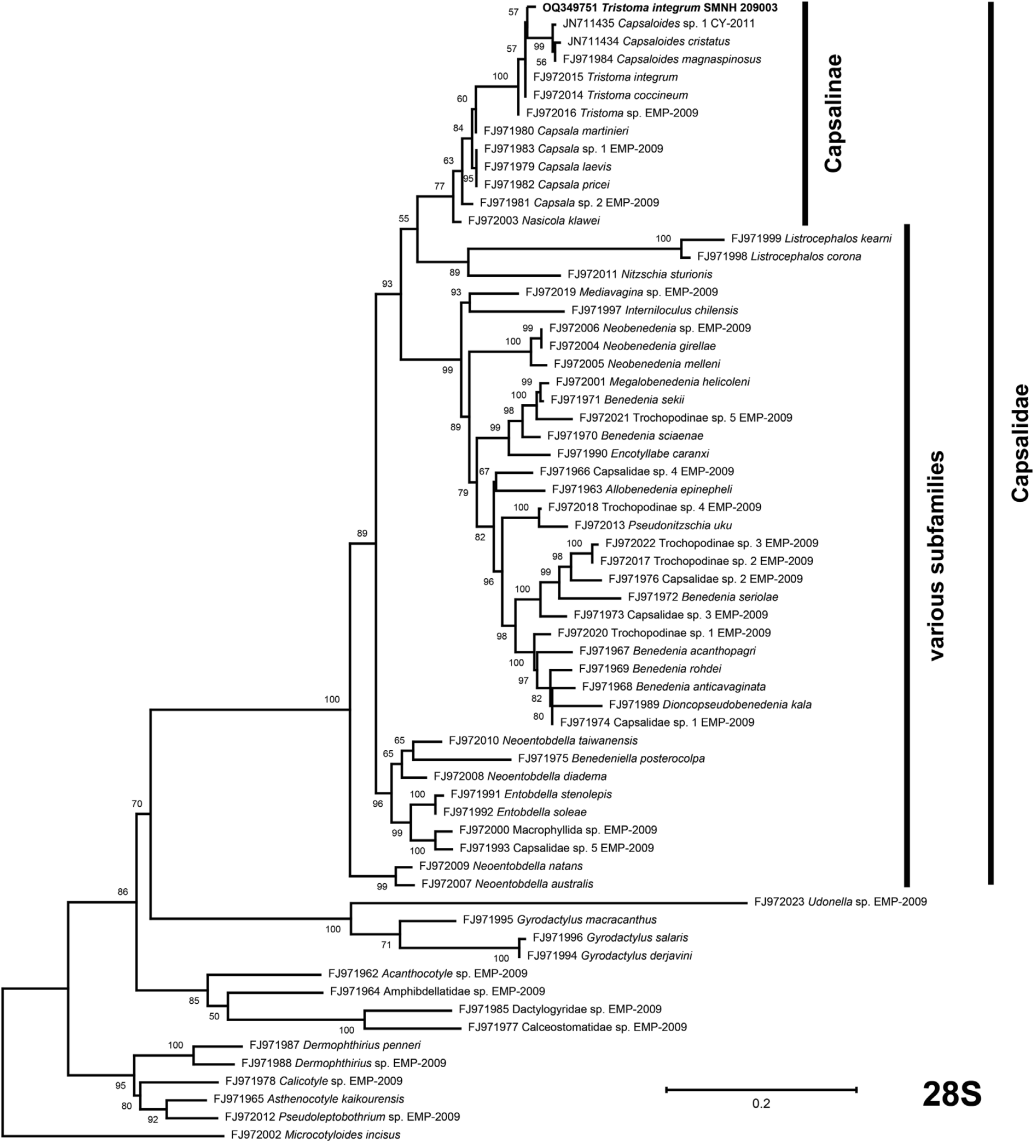
Maximum likelihood phylogenetic tree (TVM+I+G4 model) obtained from SSU (28S) partial sequences; mainly based on the data used by Perkins *et al.* (2009) [48] with the addition of several new sequences, including our new sequence of *Tristoma integrum*. The tree with the best likelihood is shown and ML bootstrap support values are indicated. The scale indicates the number of substitutions per site. The subfamily Capsalinae is indicated on the right. All other clades are a mixing of several subfamilies traditionally recognised by morphology and subfamilies are thus not indicated.

For the partial *28S* inferred phylogeny, the best model of evolution returned by ModelTest-NG was the TVM+I+G4 model. *Tristoma* appeared non monophyletic since some members of *Capsaloides* were nested within its clade. The phylogeny reflected the polymorphisms detected in the *28S* gene between *T. integrum* SMNH 209003 and other specimens of the genus. Sister-groups to the *Tristoma* clade were several members of *Capsala* and *Nasicola klawei* (Stunkard, 1962) Yamaguti, 1968 (Fig. 7). All these are members of the Capsalinae.

### Other markers available for members of the genus *Tristoma*

The complete histone 3 and elongation factor 1 genes were also obtained from *T. integrum* SMNH 209003, based on the assembly made with k-mer 85.

The intronless histone 3 gene consisted of a 2 695 bp contig with a coverage of 70.4x. The gene itself, as deposited in GenBank (OQ354712), is 411 bp long. A megablast query on the whole gene returned as best result a partial 258 bp fragment obtained from *Pseudonitzschia uku* Yamaguti, 1965 (FJ972129). Manual alignment with MEGA X of the complete gene with the partial 148 bp fragment obtained by Perkins *et al.* [48] from *T. integrum* (FJ972131) also displayed 100% identity.

The *EF1α* gene was found inside a 1 693 bp contig (GenBank: OQ349179) with a coverage of 4.8x. The gene itself was 1 476 bp long, and contained 2 group I introns (39 bp and 72 bp), leading to a 1362 bp complete coding sequence. A megablast query returned as best result a partial 492 bp long fragment obtained from *T. coccineum* Cuvier, 1817 (FJ972071), with 98.18% identity. *Tristoma coccineum* was the only species of the genus *Tristoma* for which this gene was available in GenBank.

### Host DNA and identification

From the pool of contigs obtained after assembly with k-mer 125, it was possible to retrieve an 11 425 bp contig with a coverage of 29.35x, which contained the complete cluster of rRNA of the host, *X. gladius*. After verification with Rfam and trimming, the complete cluster was 6199 bp long (GenBank: OQ349752), distributed as 1 838 bp (*18S*), 453 bp (*ITS1*), 154 bp (*5.8S*), 490 bp (*ITS2*), and 3264 bp (*28S*). A Megablast query returned as the best result a 3947 bp fragment attributed to the predicted *28S* gene of *X. gladius* (GenBank: XR_005706970), with 99.69% identity. Alignment with the 656 bp partial 28S gene of *X. gladius* used for datamining showed 100% identity between both.

Using the pool of contigs obtained with assembly k-mer parameter of 125, it was hardly possible to find traces of the host’s mitochondrial genome. However, when datamining the results obtained with k-mer 85, 26 contigs accounting for 10 568 bp were found, with an average coverage of 3.7x. By merging several of these contigs, it was possible to obtain a 1 274 bp partial *cox1* gene. Megablast query returned 99.29% identity with two full mitogenomes of *X. gladius* (AB470301 and AP006036) and 99.21% identity with two complete *cox1* genes of *X. gladius* (HM071014 and GQ202122).

## Discussion

### Identification of our specimens as *Tristoma integrum*

Given the organisation of the haptor, testes and ovary, our specimens are members of Capsalinae Johnston, 1929. By the unbifid posterior rays of the haptor and the shape of the dorsomarginal spines, they are members of *Tristoma* Cuvier, 1817 [51]. Currently, *Tristoma* includes four valid species all described from *Xiphias gladius*: *T. coccineum* Cuvier, 1817; *T. integrum* Diesing, 1850; *T. adcoccineum* Yamaguti, 1968 and *T. adintegrum* Yamaguti, 1968 [19, 24, 60]; however, we follow Chisholm & Whittington (2007) [18] and consider that the two species described by Yamaguti are probably junior synonyms.

Euzet & Quignard (1961) remarked that the oncomiracidium was identical in both species *T. integrum* and *T. coccineum* [27]. They questioned the duality of the species in *Tristoma* and hypothesised that the morphological differences between the adults of the two species could be phenotypical differences due to their different positions on the gills. The availability of sequences from both species, which are different [48], now contradicts this hypothesis.

*Tristoma integrum* and *T. coccineum* are morphologically similar but can be distinguished by the number and organisation of spines and cuspids per spines, according to Price (1939) [51]. The rows of spines on *T. integrum* are numerous, while there are fewer rows of spines on *T. coccineum*. Additionally, in *T. integrum* the spines are similar in terms of cuspids per spine, whereas in *T. coccineum* the spines are dissimilar, especially with the most median spines having only one cuspid, and the outermost ones having 10 or more cuspids and the other having 2 to 7 cuspids [51]. Our specimens correspond well to *T. integrum* (Table 2), but we noted that spines had a slightly higher number of cuspids than the specimens described by Price. The description by Price [51] was on only two specimens.

Several hosts for *Tristoma* spp. have been mentioned in addition to *X. gladius* – these probably need verification, possibly with molecular tools.

### Molecular results

#### Relevance of the protein multigene for the phylogeny of the Capsalidae

The tree (Fig. 6) shows a robust branch which includes only members of the Capsalinae. The Capsalinae includes only 4 genera (*Capsala*, *Capsaloides*, *Tristoma* and *Nasicola*) [18, 48] so this tree, which includes 3 of the 4 genera, may be considered as providing significant information for this subfamily. The tree clearly shows the monophyly of the Capsalinae. Within this branch, *Capsala* is not monophyletic, with *Capsala katsuwoni* placed as an outgroup to all other capsaline. The rest of the species included in the tree are all members of the Benedeniinae, and the tree fails to recover the monophyly of this subfamily, with *Neobenedenia melleni* placed as sister-group of the Capsalinae. It should be noted that the tree includes members of only two subfamilies among nine recognised in traditional classifications (Perkins *et al.*, 2009, their Table 1 [48]), and that the interest of our results is therefore limited. However, Perkins *et al.* (2009), in a molecular study based on various markers, noted that the single subfamily they could retrieve as monophyletic was the Capsalinae [48]; we therefore confirm their conclusion.

#### Relevance of the 28S tree for the phylogeny of the Capsalidae

The tree (Fig. 7) includes many more taxa than the protein tree. A major result is the monophyly of the Capsalinae, with representatives of all four genera of the subfamily (*Capsala*, *Capsaloides*, *Tristoma* and *Nasicola*) united together in a strong monophyletic clade. For all other subfamilies, results are much less in accordance with traditional taxonomy. A clade containing a Nitzschiinae and two Entobdellinae (*Listrocephalos* spp.) is sister-group to the Capsalinae. Three other branches appear to have robust support, but each of them is a mix of Trochopodinae, Benedeniinae and Entobdellinae, therefore precluding any comparison with phylogenies and classifications based on morphology. Again, this reminds us of the results of Perkins *et al.* (2009) [48] on a smaller taxonomical sampling, who concluded that they could only retrieve monophyly for the Capsalinae. We note that our limited results based on mitogenome proteins also found the Capsalinae monophyletic. The Capsalinae are all parasites of gamefish [18, 48] and it is likely that they have evolved within this group of pelagic, high-speed animals.

## Conflict of interest

The Editor-in-Chief of Parasite is one of the authors of this manuscript. COPE (Committee on Publication Ethics, http://publicationethics.org), to which Parasite adheres, advises special treatment in these cases. In this case, the peer-review process was handled by an Associate Editor, Jérôme Depaquit.

## Figures and Tables

**Figure A1:**
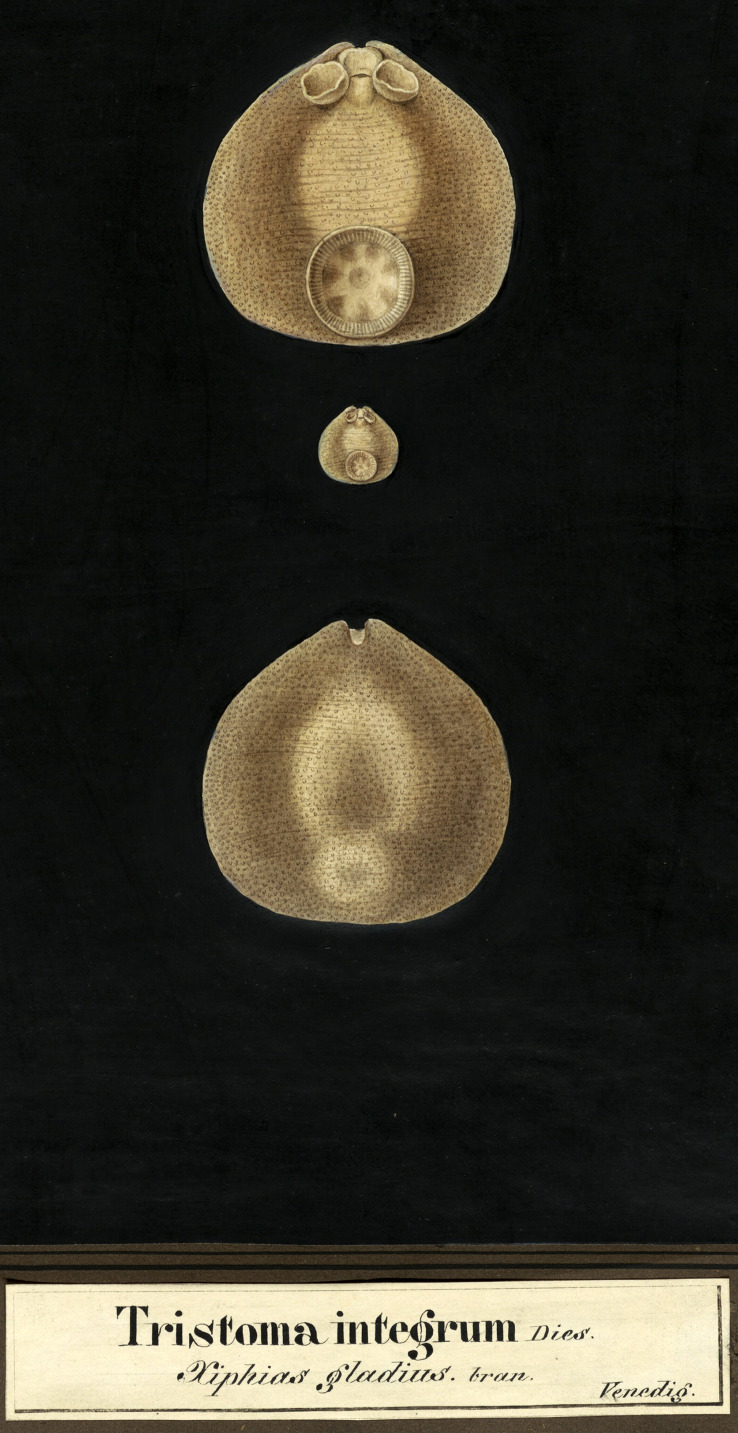
The drawing of *Tristomum integrum* by Joseph Zehner, 1845. The label of the drawing reads “*Tristoma integrum* Dies., *Xiphias gladius* bran., Venedig”, and no text is associated with it. Reproduced with authorisation from the original now kept in the Austrian National Library, digitised and publicly available as https://onb.digital/result/1176CD51.

## Appendix

### Historical account and intricate nomenclature of *Tristoma* and *Tristoma integrum*

Cuvier (1817, pages 40–43 [19]) provided a general classification of the “Trématodes” (i.e., species which would be now classified within the Digenea, Aspidogastrea and Monogenea). Apparently, the only monogeneans known to him were the Polystomes, with the only genus *Polystoma* Zeder (now in the Polystomatidae) and the “Tristome” (note the singular), proposed as *Tristoma* Cuvier, with a single species, *Tristoma coccineum* Cuvier, which attaches to the gills of several fishes of the Mediterranean (“la *môle*, le *xiphias*, etc.”). The description includes an anterior large cartilaginous sucker and two smaller posterior suckers, i.e., Cuvier misinterpreted the haptor as anterior. No etymology was given, as was usual at that time, but we can safely interpret that *Tri-stoma* referred to the presence of three mouths. Such an error in the interpretation of the orientation of the body and the designation of suckers as several mouths is found in other monogeneans described in the nineteenth century, such as *Hexostoma* Rafinesque, 1815 (see Ayadi *et al.*, 2022 [2]) or *Polystoma* Zeder, 1800 (images oriented with suckers at the top in Zeder, 1803 and van Lidth de Jeude, 1829 [57, 64]). The singular for “Tristome” in Cuvier’s text shows that he considered that a single species of monogenean, apart the Polystomes, was known to him at that time; however, Cuvier [19] mentioned in the references a similar species on the “Diodon” that was found by Lamartinière (cited as “Journal de Physique” 1797 in Cuvier – we could not retrieve this publication), but did not cite the formal description of this animal by Bosc as *Capsala martinieri* Bosc, 1811, within the genus *Capsala* Bosc, 1811 [10]. It should be noted that Lamartinière classified this animal under the general designation of “insect” and that Bosc [10] considered it a crustacean, however noted that crustaceans have legs, but not this species.

Dujardin (1845, page 323) mentioned “I could verify several details of the structure of this helminth [*Tristoma coccineum*] in the specimens in the Paris Museum” [26]. Dollfus (1968) published and commented unpublished documents left by Dujardin [25]. He wrote (Dollfus, 1968, pages 122–123 [25]) that the specimen described by Dujardin had a body with a profound posterior incision and thus was not *T. coccineum*. Dollfus added that no hand-written note about *Tristoma* could be found in Dujardin’s documents, and that the type specimen described by Cuvier “had not been found until this date” (1968) in the collections of the MNHN in Paris. We confirm (2023) that this is still the case.

Diesing (1835) provided a monography of *Tristoma*, with its German text fully translated into French published in 1836 [22, 23]. He considered that other genera such as *Capsala*, *Phylline,* Oken 1815, *Hirudinis* Abilgaard, 1794 and *Nitzschia* Baer, 1826 were junior synonyms of *Tristoma*, and therefore included within the latter genus a total of five species, among which only *T. coccineum* is still considered a member. Diesing [22, 23] followed Rudolphi (Rudolphi, 1794, publication not seen) and corrected the error of earlier scientists, considering the haptor as posterior.

Diesing (1850, pages 428–431 [24]) provided a list of species within *Tristoma*, here spelled *Tristomum*. “*Tristomum integrum”* Diesing was only cited as a synonym of “*Tristomum coccineum*” Cuvier with the cryptic indication “in Collect. Zoograph. Ferdinandi I. Imperatoris”. Some research allowed us to discover that this designates a collection of drawings which were discussed in a footnote in Spengel, 1912 [55], here translated from the original German: On November 19, 1910, F. Siebenrock wrote to Prof. Taschenberg in Halle about this work, which was cited several times by Diesing and after him by Greeff: “The work is in the entailed library of the imperial family. It consists of a collection of pages with hand drawings made by a Viennese draftsman on behalf of Emperor Ferdinand I. A text is completely missing from the work, only the name is attached to the individual figures. If the professor intends to obtain more precise data from the work, please contact the librarian and board member of the k. k. Family Fideikomiss Library of the Supreme Imperial House of Dr. F. Schnuerer in Vienna, I/I, Burgring, Neue Hofburg”. Spengel added: “Since the ‘Icones’ were not published, they do not come into consideration as a ‘publication’”. From the above, the conclusion is that Diesing (1850) considered his species *T. integrum* as a junior synonym of *T. coccineum* and that the drawing of the species was in an unpublished work.

We sought the help of Dr. Helmut Sattmann, recently retired as Curator at the Natural History Museum of Vienna, who kindly checked the historical drawings available in the invertebrate collection of the Natural History Museum of Vienna. He found that no drawing was labelled “*Tristomum integrum*” in this Institution. After some research, he informed us that the drawing of *T. integrum* was recently digitised by the Austrian National Library and was available on the internet from https://onb.digital/result/1176CD51. The drawing is catalogued with the author name and date: Zehner, Joseph [MalerIn], 1845. Its label reads: “*Tristoma integrum* Dies., *Xiphias gladius* bran., Venedig [German for Venice]”. Dr. Helmut Sattmann commented “I tend to assume that the museum owns some originals and those were provided for scientific publication purposes that time. e.g., *T. coccineum*, published by Bremser 1824, is present in the museum’s collection. My conclusion is, that *T. coccineum* is in the museum’s collection, because it was used for publication, but *T. integrum* is in the national library, because it was not used for publication (neither by Bremser nor by Diesing).”

The drawing by Joseph Zehner now digitised in the collections of the Austrian National Library has probably never been seen by parasitologists since the middle of the nineteenth century. We are showing it here (Fig. 8).

In view of the above, it is somewhat surprising, for a taxonomist accustomed to modern editions of the International Code of Zoological Nomenclature, that the species *T. integrum* could be considered available. The reason for this is certainly the complex reasoning made by Price (1936, 1939). Price (1936, summary of his thesis; 1939, full publication [50, 51]) re-evaluated several species described in the nineteenth century, including *Capsala martinieri*, for which he provided a copy of the original description in French and of the drawings by Lamartinière (1797) and a description of new specimens [51]. For *Tristoma*, he considered that *T. coccineum* was valid. He discussed various interpretations of *T. integrum* and *T. coccineum* by several authors (Blanchard, Risso, Diesing, and Taschenberg) and finally concluded (Price, 1939, page 86 [51]) that “the species described by Taschenberg as *T. coccineum* must be regarded as a separate species and take the oldest available synonym which is *Tristoma integrum* Diesing”. Sproston (1946) noted for *T. integrum* “Diesing, 1850, 429 (apparently a MS. Name, as synonym of *T. coccineum* Cuv.; Price, 1936, 12: revives the species name of Diesing)”, and finally noted “the validity of *Tristoma* spp. is in great need for revision”.

The Code [32] differentiates works before 1961 from those published during and after 1961: “Article 11.6. Publication as a synonym. A name which when first published in an available work was treated as a junior synonym of a name then used as valid is not thereby made available. 11.6.1. However, if such a name published as a junior synonym had been treated before 1961 as an available name and either adopted as the name of a taxon or treated as a senior homonym, it is made available thereby but dates from its first publication as a synonym”. From the above, we conclude that Price (1936 [50]) acted as first reviser (Article 24 of the ICZN [32]) and that his decision to use a name first proposed as synonym is acceptable (Article 11.6.1). Finally, *Tristoma integrum* Diesing, 1850 is available according to the code. We therefore use Price (1939) for distinguishing the two species *T. integrum* and *T. coccineum*.

There is some irony here in that the figure made by Joseph Zehner in 1845 and reproduced here as our Figure A1, which cost us and several helpful colleagues some efforts to locate, has no relevance for systematics or nomenclature, as the decisions by Price were solely on the name and not on his examination of the figure.
